# Classroom Temperature and Learner Absenteeism in Public Primary Schools in the Eastern Cape, South Africa

**DOI:** 10.3390/ijerph182010700

**Published:** 2021-10-12

**Authors:** Vicky Pule, Angela Mathee, Paula Melariri, Thandi Kapwata, Nada Abdelatif, Yusentha Balakrishna, Zamantimande Kunene, Mirriam Mogotsi, Bianca Wernecke, Caradee Yael Wright

**Affiliations:** 1 Department of Environmental Health, School of Behavioural and Lifestyle Sciences, Faculty of Health Sciences, Nelson Mandela University, Gqeberha 6019, South Africa; pulevicky@gmail.com (V.P.); angela.mathee@mrc.ac.za (A.M.); paulaezinne.melariri@mandela.ac.za (P.M.); 2 Environment and Health Research Unit, South African Medical Research Council, Johannesburg 2094, South Africa; thandi.kapwata@mrc.ac.za (T.K.); zamantimande.kunene@mrc.ac.za (Z.K.); mirriam.mogotsi@mrc.ac.za (M.M.); bianca.wernecke@mrc.ac.za (B.W.); 3 Environmental Health Department, Faculty of Health Sciences, University of Johannesburg, Johannesburg 2094, South Africa; 4 Biostatistics Research Unit, South African Medical Research Council, Durban 4001, South Africa; nada.abdelatif@mrc.ac.za (N.A.); yusentha.balakrishna@mrc.ac.za (Y.B.); 5 Environment and Health Research Unit, South African Medical Research Council, Pretoria 0001, South Africa; 6 Department of Geography, Geoinformatics and Meteorology, University of Pretoria, Pretoria 0002, South Africa

**Keywords:** climate change, cold, environmental health, heat, public health, humidity, schoolchildren

## Abstract

Children spend a significant proportion of their time at school and in school buildings. A healthy learning environment that supports children should be thermally conducive for learning and working. Here, we aimed to study the relations between indoor classroom temperatures and learner absenteeism as a proxy for children’s health and well-being. This one-year prospective study that spanned two calendar years (from June 2017 to May 2018) entailed measurement of indoor classroom temperature and relative humidity, calculated as apparent temperature (Tapp) and collection of daily absenteeism records for each classroom in schools in and around King Williams Town, Eastern Cape province, South Africa. Classroom characteristics were collected using a standardized observation checklist. Mean indoor classroom temperature ranged from 11 to 30 °C, while mean outdoor temperature ranged from 6 °C to 31 °C during the sample period. Indoor classroom temperatures typically exceeded outdoor temperatures by 5 °C for 90% of the study period. While multiple factors may influence absenteeism, we found absenteeism was highest at low indoor classroom Tapp (i.e., below 15 °C). Absenteeism decreased as indoor Tapp increased to about 25 °C before showing another increase in absenteeism. Classroom characteristics differed among schools. Analyses of indoor classroom temperature and absenteeism in relation to classroom characteristics showed few statistically significant relations—although not exceptionally strong ones—likely because of the multiple factors that influence absenteeism. However, given the possible relationship between indoor temperature and absenteeism, there is a learning imperative to consider thermal comfort as a fundamental element of school planning and design. Furthermore, additional research on factors besides temperature that affect learner absenteeism is needed, especially in rural areas.

## 1. Introduction

A safe school location and structure as well as healthy indoor and outdoor school environments are essential elements of a space that is conducive to learning. Children and adolescents spend a significant proportion of their time at school. Healthy physical school environments may support healthy children and positive learning [1]. Environmental conditions at school may vary widely depending on geographical location, urban versus rural settings, and socio-economic variations, among other factors [1]. Some of the environmental risks include a hazardous school location, for example, beside an industrial area, inadequate water and sanitation, the presence of ambient and indoor air pollution, pesticides, unsafe food, mould, and excess solar ultraviolet radiation exposure [1,2].

One risk factor that relates to both school location and school building characteristics is indoor classroom temperature and relative humidity. Several studies have found a negative association between temperature and relative humidity versus learner achievement [3,4,5,6]. Ideal classroom conditions conducive to learning are within a narrow band of temperature and relative humidity of 20–22 °C and 50%, respectively [7]. In a meta-analysis of 18 studies [8] performance by learners on psychological tests could be expected to increase on average by 20% if classroom temperatures were lowered from 30 to 20 °C. Optimal performance was achieved at temperatures lower than 22 °C; however, these studies were all conducted in temperate climates, and hence, validation in other climates, such as sub-tropical climates, is needed.

Sustainable Development Goal (SDG) 4–Quality Education calls for safe, healthy environments in which learners can learn and work to ensure quality education [9]. In many schools around the world, classroom conditions are not conducive to facilitating safe learning spaces. The health impacts of unhealthy school environments can also affect other outcomes beside academic performance of learners. School building characteristics such as poor ventilation, air pollution, the presence of vermin, and visible mould also influence learner absenteeism [10,11,12,13]. In a study of Upstate New York school building conditions compared to New York State Education Department learner absenteeism data, learner absenteeism was associated with high relative humidity (odds ratio: 3.07, 95% Confidence Interval (CI): 1.37, 6.89) after adjusting for confounding factors [14]. However, this was not the case in Scottish schools where absenteeism was not affected by indoor classroom relative humidity or temperature [15]. This variation among studies points to the need for country-specific, baseline data to assess the relations between indoor temperature and relative humidity on learner absenteeism. Furthermore, in the context of climate change and global warming, indoor classroom temperatures in buildings that mimic ambient (outdoor) temperatures may experience changes in indoor classroom temperatures if the appropriate interventions are not put in place.

South Africa is expected to experience an average increase of 4 to 6 °C in ambient temperatures by the end of the 21st century [16]. In school buildings with natural ventilation, no insulation, and built from materials that poorly maintain a constant indoor classroom temperature, the warming of ambient temperatures likely leads to the warming of indoor classroom temperatures. Similarly, if night-time temperatures decrease, for example from a decrease in cloud cover and more clear skies, early morning temperatures inside classrooms may decrease. Maintaining thermal comfort in such school buildings will become a challenge in a changing climate [17].

Few studies have been conducted in South Africa to assess the impact of indoor classroom temperatures on learners’ health and/or academic performance. A Johannesburg study (in Gauteng province) found that learners felt tired, had low concentration, and felt sleepy when temperatures were hot during the school day [18]. Moreover, there was a statistically significant correlation, when controlling for school cluster effect and time of day, between indoor classroom temperatures ≥32 °C and learners who felt tired and found it difficult to breathe. School building characteristics contributed to indoor classroom temperatures—consistently higher temperatures were seen in classrooms constructed of prefabricated asbestos sheeting, corrugated iron roofs, and converted shipping containers [18]. Such information is useful for informing school building design with a focus on learner health and achievement as well as in the context of climate change.

Given the abovementioned concerns, this study assessed the relationship between indoor classroom temperature and learner absenteeism in the Eastern Cape province of South Africa. To meet the study’s aim, three objectives were set: (1) to measure indoor classroom and outdoor temperature and relative humidity, as well as learner absenteeism for one year; (2) to assess relations between temperature and relative humidity and learner absenteeism for the same year; and (3) to assess classroom building characteristics that may affect the relations between indoor temperature and learner absenteeism. This information would be informative in the design and/or retrofitting of school classrooms in relation to classroom temperature. Since it is acknowledged that multiple factors may influence learner absenteeism, the study also included a questionnaire sent home to parents/guardians to try and understand socio-demographic-economic factors that could influence absenteeism.

## 2. Materials and Methods

### 2.1. Study Design

The study was designed as a prospective study in government schools to assess classroom temperatures in relation to learner absenteeism. The South African school calendar runs from January to December. The one-year study began mid-year (i.e., June 2017) due to delays in approvals; hence, learners in Grade 3 who worked in selected classrooms were included in the study for the first six months. At the beginning of the following year, new Grade 3 learners in the same selected classroom were enrolled in the study until May 2018. In that way, one year of data was obtained for the study. One reason for why Grade 3 learners were chosen for the study was because they spend all their school day (from ~7 h 30 to 13 h 30) in the same classroom and do not move between classrooms. Other reasons are given in Section 2.3 below.

### 2.2. Study Area

**Figure 1:**
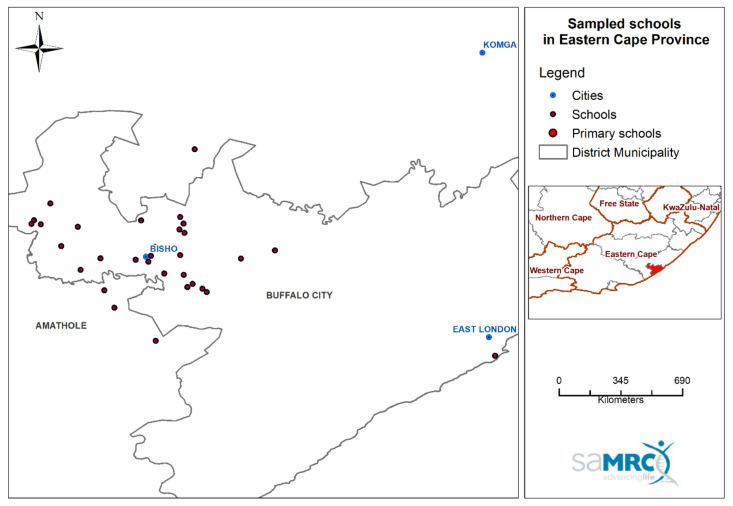
Location of the schools that participated in the study (map drawn by T.K.).

The study was conducted in and around King Williams Town in the Eastern Cape province of South Africa (Figure 1). The typical weather in King Williams Town is mild with summertime (December–February) and wintertime (June–August) minimum and maximum temperatures of 16 and 27 °C, and 7 and 21 °C, respectively. The town experiences both winter and summer rainfall receiving 350–600 mm of rainfall per annum. The Eastern Cape is a relatively underdeveloped province and is home to about 6.5 million people, of whom 49% live in rural settings [19].

### 2.3. Study Sample and Procedures

In this study, 30 public (i.e., government) primary schools were randomly selected from the public database of the Department of Basic Education to participate from a total of approximately 271 schools in the King Williams Town Education District. At each school, one Grade 3 classroom that predominantly faced north was selected to try and capture worst-case scenarios for warm and cold temperatures in a southern hemisphere setting. Grade 3 learners were considered an appropriate target grade since children aged 8–9 years old are unlikely to smoke, consume alcohol, or be truant. They also remain in the same classroom during the school day.

A researcher contacted and visited the school principal to obtain permission to conduct the study at their school. Following agreement, the researcher met with the teacher of the selected classroom to request assistance with implementing the study protocol. Study dates were agreed upon for the installation of temperature/relative humidity dataloggers. Temperature/relative humidity and absenteeism data were collected for one year from 30 June 2017 to 31 May 2018. The loggers were checked by the research team every three months, and data were downloaded to a password-protected computer. Unique identifier codes were used for schools to ensure anonymity and compliance with the Protection of Personal Information Act [20]. Approvals were obtained from the Provincial Department of Education, chairpersons of the school governing bodies and school principals, parents/guardians (via informed consent). Research ethics clearance was granted by the Nelson Mandela University Research Ethics Committee (H-16-HEA-ENV-002).

### 2.4. Absenteeism Data

Absenteeism data were collected directly from each of the schools using a daily attendance sheet. Data were in the form of daily records (or counts) of absent learners. These records were manually entered into Microsoft Excel as absenteeism counts per day. Double data entry was performed by the researcher and the primary investigator of the study to ensure data accuracy. All data were anonymous, and no personal information was captured in the electronic form for the absenteeism data.

### 2.5. Learner’s Parent/Guardian Questionnaire

A questionnaire was sent home with learners for their parents or guardians to complete. The questionnaire included two sections: household characteristics (i.e., anyone with a social grant; anyone having medical aid; dwelling type; primary energy and water sources; and sanitation); and learner-related school variables (numbers of learners attending schools of different quintiles; distance to school; school feeding programme present; and general reasons for absence from school). These records were coded and manually captured in Microsoft Excel [21]. Double data entry was performed by the researcher and the primary investigator of the study to ensure data accuracy. All data were anonymous, and no personal information was captured in the electronic form for the questionnaire data. Learner sample description data were analysed using Stata version 16.1 [22].

### 2.6. Temperature and Humidity Measurements: Indoor Classroom and Outdoor (Ambient)

iButton/Lascar temperature and relative humidity dataloggers were used to measure indoor classroom temperature and relative humidity. The loggers were mounted out of sight at a height of approximately 2 m above the learners’ heads and away from windows and direct sunlight. Loggers were set to record every 10 min and were activated on continuous mode from date of installation to date of retrieval. The 10 min readings were averaged to obtain hourly average temperature and relative humidity values. The school day starts around 7 h 30 and ends around 13 h 00 or 13 h 30; hence, the data for those periods of the day when the children were in the classroom were included in the study. Outdoor temperature and relative humidity, as well as windspeed data (required for the apparent temperature calculation), were obtained from the nearby South African Weather Service’s (SAWS) Bhisho Weather Station.

In addition to temperature, we calculated apparent temperature (Tapp) using the measurements made by the loggers for the classrooms and the SAWS data. Tapp is an indicator of thermal sensation or “real-feel’ temperature [23]. Tapp was calculated using Equation (1) as follows:

Tapp = Ta + 0.33 × e − 0.70 × ws − 4.00(1)

where Tapp is apparent temperature, Ta is measured dry bulb temperature (℃) in the classroom, e is water vapour pressure (hPa), and ws is wind speed (set to 0 for indoors and using wind speed data from SAWS for outdoor conditions) [23]. Water vapour pressure was calculated using the humidity measurements made in the classrooms and applying Equation (2) as follows:

e = rh/100 × 6.105 × exp (17.27 × Ta/(237.7 + Ta))(2)

where rh is relative humidity (%). The Tapp data were applied in the statistical modelling of absenteeism and indoor classroom temperatures, since real-feel temperature has been associated with health symptoms and impacts (see Table 1) [24]. Measured indoor classroom temperatures calculated as Tapp are discussed considering these thresholds.

### 2.7. School Classroom Characteristics Observation Checklist

The fieldworker observed the classroom characteristics in which the temperature and relative humidity measurements were made using an observation checklist. Selections were made for seven characteristics: type of roof, type of floor, exterior material of classroom wall, interior material of classroom wall, presence or absence of window shade, presence or absence of a ceiling, and presence or absence of temperature control in the classroom.

### 2.8. Data Processing and Statistical Analyses

Temperature and relative humidity data were downloaded from the dataloggers directly to a computer via USB and saved in a .txt file. Absenteeism data were collated in Microsoft Excel. Classroom characteristics were coded and entered in Microsoft Excel [21]. All data were merged into a single database using Stata version 16.1 [22].

Statistical analyses were performed using Stata version 16.1 [22] and R version 4.0.2 [25]. These analyses included descriptive statistics of the classroom and school characteristics as well as time-series plots exploring daily and seasonal variations in temperature and Tapp.

A Mixed Effects Poisson Model (MEPM) was run and compared to a Mixed Effects Negative Binomial Model (MENBM) to determine which model had the better fit using the likelihood ratio test. MENBM is used when overdispersion is present in the data, more than would be expected when using MEPM. The MENBM produced a better fit and was used to determine the effects of classroom indoor temperature (Tapp was not used here since for practical reasons in terms of school policy development and classroom design, and temperature is easier to apply than a calculation such as Tapp), and classroom characteristics on learner absenteeism, with school included as a random effect.

## 3. Results

### 3.1. Sample Descriptives

**Figure 2:**
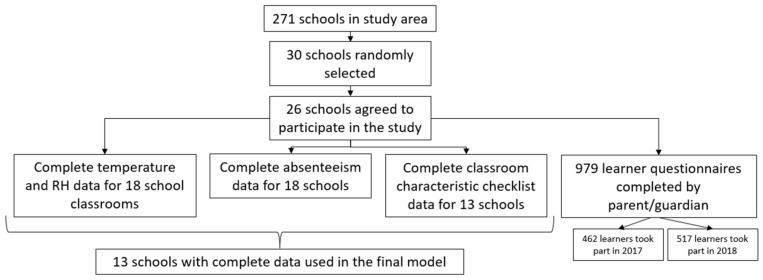
Flow chart indicating data completeness for different study instruments. Note. RH: relative humidity.

Of the 30 schools randomly selected from the study area, 26 schools agreed to participate, and indoor temperature and relative humidity were logged in the selected Grade 3 classroom at each school for one year. Complete datasets for temperature and relative humidity and learner absenteeism data were only available for 18 schools due to lack of completeness of data from some schools (Figure 2).

Classroom characteristic checklist data were complete for 13 schools; therefore, the final model focussed on classroom temperature and learner absenteeism as an outcome incorporating data from these 13 schools. The learner questionnaire was completed by 462 and 517 parents/guardians in 2017 and 2018 (total *n* = 979), respectively. This represented a 55% (*n* = 1777) response rate for the learner questionnaire completed by parents/guardians.

Table 2 presents the descriptive findings of learner questionnaire data for the learners enrolled in the study. These data were used in the discussion to interpret the model results. School quintile relates to a five-tier system that determines the government’s budget allocation to a school. Quintiles one–three (considered the poorest schools) are ‘no fee paying’ schools and quintiles four–five (the least poor schools) are ‘fee paying schools’. Quintile-poverty rankings are determined by considering the level of poverty among the community around the school and certain infrastructural factors [26].

### 3.2. Indoor Classroom Temperature and Outdoor Temperature

**Figure 3:**
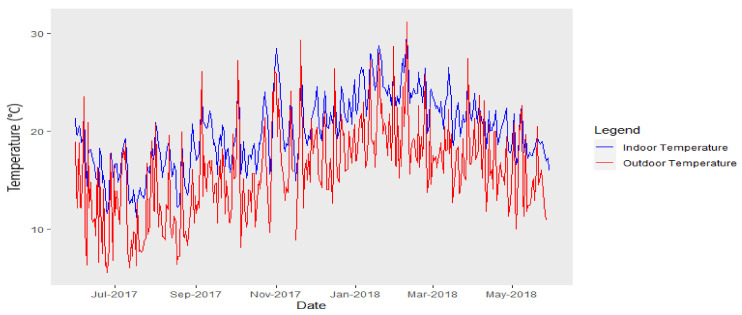
Mean temperature (°C) measured inside classrooms (blue line) and outside classrooms (red line) for all schools (*n* = 18 schools).

During the study period, mean measured indoor classroom temperature ranged from 11 to 30 ℃, while mean outdoor temperature ranged from 6 to 31 °C (Figure 3). Both indoor classroom and outdoor temperature displayed seasonal patterns with highest daytime temperatures being observed during summer months and lowest temperatures during winter months.

Indoor classroom temperatures typically exceeded outdoor temperatures by a mean of 5 °C, and this indoor classroom percentage exceedance of outdoor temperature occurred on 93% of days during the full study period.

**Figure 4:**
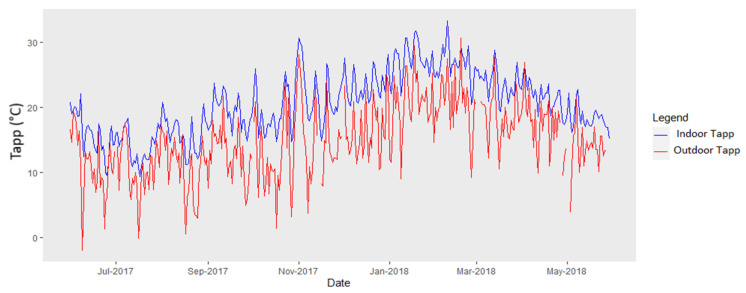
Mean Tapp (°C) calculated for inside classrooms (blue line) and outside classrooms (red line) for all schools combined (*n* = 18 schools).

Outdoor Tapp (i.e., real-feel temperature) ranged from −2 to 31 °C (Figure 4) and indoor Tapp ranged from 9 to 33 °C. Tapp levels exceeded the US NWS classification [24] symptom bands I (Caution) and II (Extreme caution) during the late spring, summer, and early autumn months.

### 3.3. Absenteeism and Indoor Classroom Apparent Temperature

While 26 schools agreed to participate, it was only possible to collect absenteeism data from 18 participating schools for the class of learners who worked in the classroom where temperature and relative humidity levels were being logged. It was not possible to collect absenteeism data from the remaining seven schools due to several challenges, e.g., lost paperwork, change in teacher, etc. There was a total of 2334 absent person-days (the total number of days in which those learners were absent combined with the number of learners absent each day) during the one-year study period.

**Figure 5:**
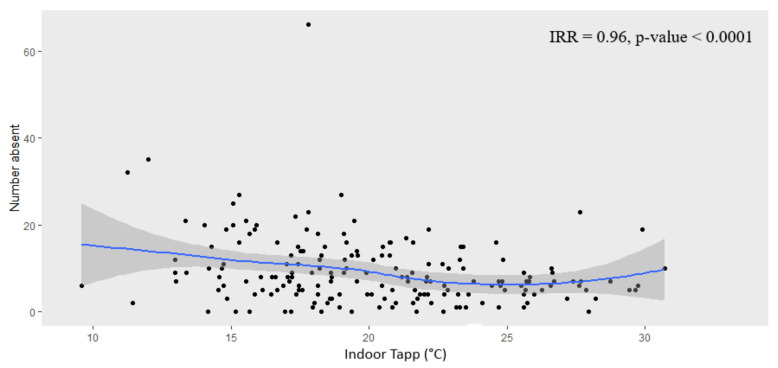
Learner absenteeism (counts) versus mean indoor classroom Tapp (°C) for all schools. The blue line is the trend line, and the grey shaded area is the confidence interval. Note. IRR: Incidence Risk Ratio.

Absenteeism appeared to be highest at low indoor classroom Tapp (i.e., below 15 °C) (Figure 5). Absenteeism decreased as indoor Tapp increased to about 25 °C before showing another increase in absenteeism. A similar finding occurred for outdoor Tapp, however, without the increase in absenteeism from 25 °C.

**Figure 6:**
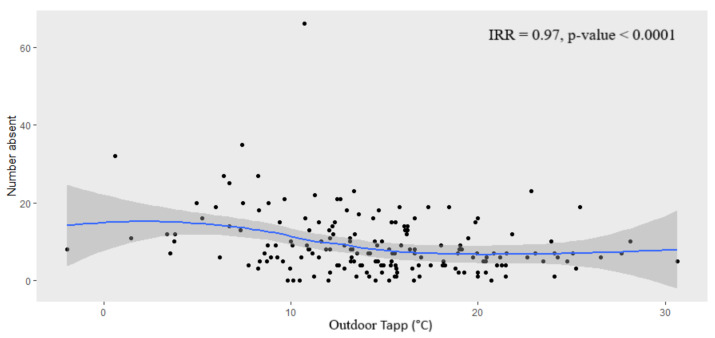
Learner absenteeism (counts) versus mean outdoor Tapp (°C) for all schools. The blue line is the trend line, and the grey shaded area is the confidence interval. Note. IRR: Incidence Risk Ratio.

With regards to the ‘outlier’ that may be observed in Figure 6, absenteeism and indoor Tapp data for all schools were aggregated to obtain daily total counts to plot against daily indoor Tapp; therefore, the ‘outlier’ point does not represent one learner. Instead, it shows that on 10 August 2017 (noted as the day after an annual public holiday on the 9 August in South Africa) there was a total of 66 learners absent and, on that date, the average indoor Tapp of all schools was 17.8 °C (see Figure 4).

### 3.4. School Classroom Building Characteristics

To assess classroom building characteristics that may affect the relations between indoor temperature and learner absenteeism, we considered roof material, ceiling material, floor material, exterior material of school wall, interior classroom wall material, shading of windows, and any form of temperature control within the classroom.

Classroom building characteristics varied between schools (Table 3). The majority of school classrooms had metal sheeting (i.e., corrugated iron) or asbestos roofs with ceiling boards and no temperature control or shade protection over the windows.

### 3.5. Multivariate Analyses Results

Indoor classroom temperature and classroom characteristics (i.e., building material, floor, ceiling, etc.) were explored in relation to learner absenteeism (Table 4). We assessed the effects that classroom characteristics and classroom indoor temperature have with each other by including interaction effects in the model. Details about the classroom roof were not included because they did not show any model improvement.

**Figure 7:**
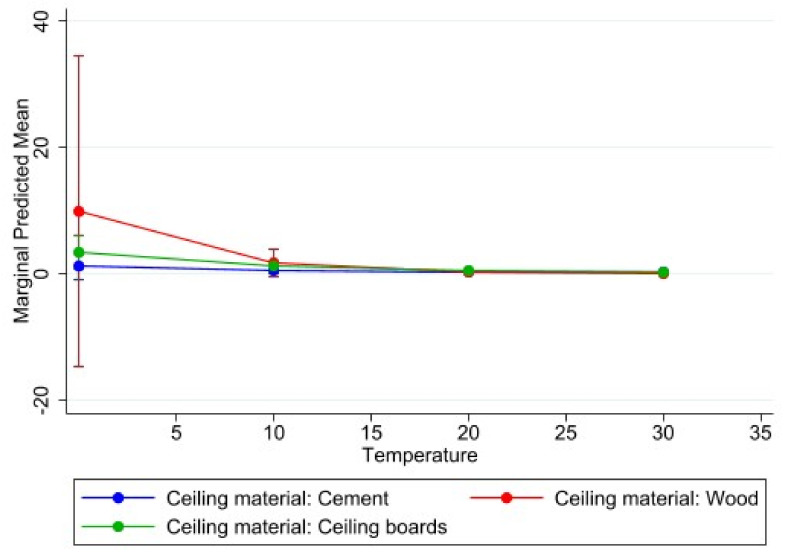
Marginal effects with 95% confidence intervals of learner absenteeism at various levels of classroom indoor temperature (°C) in relation to ceiling materials.

**Figure 8:**
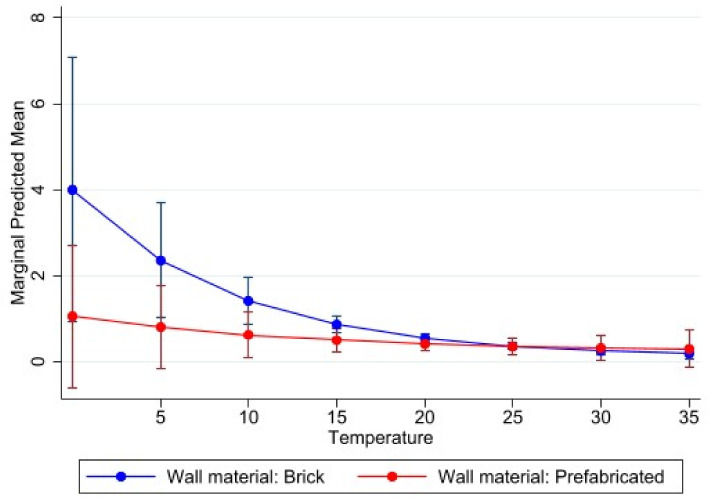
Marginal effects with 95% confidence intervals of learner absenteeism at various levels of classroom indoor temperature (°C) in relation to wall materials.

**Figure 9:**
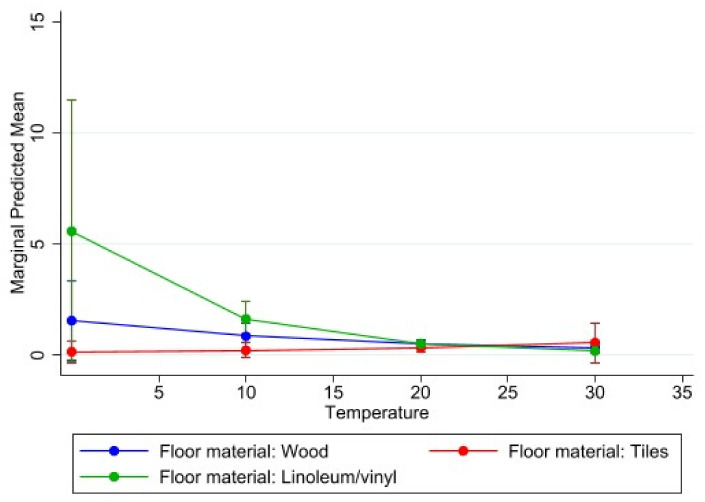
Marginal effects with 95% confidence intervals of learner absenteeism at various levels of classroom indoor temperature (°C) in relation to floor materials.

The presence of floor tiles compared to other types of flooring was found to be statistically significantly associated at the 95% confidence level with absenteeism (*p* = 0.020), as well as with the effect between classroom indoor temperature and absenteeism (*p* = 0.045). The combined effect of prefabricated schools and wooden and/or tiled floors with classroom indoor temperature, respectively, decreased with learner absenteeism, whereas the combined effects of wooden ceilings and ceiling boards with classroom indoor temperature increased with learner absenteeism (Table 4). Figure 7, Figure 8 and Figure 9 visually illustrate these relationships.

## 4. Discussion

To the best of our knowledge, this study represents the longest time series of classroom temperatures in South African schools in any province. Mean indoor classroom temperature ranged from 11 to 30 °C, while mean outdoor temperature ranged from 6 to 31 °C during the study period. These are large mean ranges. There is a need to address indoor classroom environments, shown here in our study to likely be on factor affecting learner absenteeism, a proxy for morbidity.

Indoor classroom temperatures typically exceeded outdoor temperatures by 5 °C for 93% of the study period. To put that in perspective, if a maximum temperature on a given day was 29 °C outdoors, the temperature in the classroom in which the learners were working was 34 °C.

Outdoor Tapp (i.e., real-feel temperature) ranged from −2 to 31 °C and indoor Tapp ranged from 9 to 33 °C. These Tapp levels exceeded the US NWS classification (as shown in Table 1) symptom bands I (Caution) and II (Extreme caution) during the late spring, summer, and early autumn months. This suggests that individuals, especially vulnerable groups such as learners with pre-existing diseases, may have experienced symptoms and side effects of heat when temperature and relative humidity combined as Tapp reached these levels. There are no temperature threshold guidelines set for educational/classroom settings. When compared with the United States Department of Labour occupational lower and upper limits of temperature thresholds set at 20 and 24 °C, respectively [27], our measured classroom temperatures were higher. The Canadians advise office thermal conditions for summer to be between 23 and 26 °C and for winter, 20 and 24 °C [28]. The South African Labour Guide advises indoor temperatures should be between 21 and 26 °C, with 21–24 °C and 24–26 °C for summer and winter, respectively [29].

Other African countries, such as Cameroon, also experience heat in school classrooms. Among 285 schoolchildren aged 12–16 years, self-reported fatigue, feeling very hot, and headaches ranged between about 20% and 76% depending on the health outcome [30]. In an Indian study that considered thermal comfort in classrooms, summertime temperatures were high (i.e., 33 °C), yet students seemed to adapt when natural ventilation, such as opening of doors and windows, was applied [31]. The latter study considered the American Society of Heating, Refrigerating, and Air-Conditioning Engineers (ASHRAE) thermal comfort standards as being too low, and that students could adapt to warmer temperatures, although the measure of outcome for the term ‘adapt’ was not well defined [32].

We found that absenteeism appeared to be highest during the periods of low indoor classroom Tapp (i.e., below 15 °C). Absenteeism decreased as indoor Tapp increased to about 25 °C before showing another increase in absenteeism. While there are no thermal comfort guidelines for South African schools, considering the occupational thermal exposure limits, there is evidence for concern among schools in the Eastern Cape province, where classroom temperatures are exceeding thresholds for optimal health and safety.

Analyses of indoor classroom temperature and absenteeism in relation to classroom characteristics showed few statistically significant relations and not exceptionally strong ones (see study limitations). Interestingly, when looking only at floor material and absenteeism, we saw a relationship between tiled floors and less absenteeism. However, when the model took indoor classroom temperature into account in this relationship, tiles were linked with a slight increase in absenteeism (with cement as the reference category). Tiles and cement floors are quite similar in their thermal characteristics. Similar findings were seen in coastal community dwellings in the Eastern Cape province, where dwellings (not classrooms) with cement floors were cooler than dwellings with any other floor type [33].

School studies elsewhere (mainly in Europe, hence temperate climates dissimilar to South Africa) have considered classroom thermal conditions and cognitive testing (not absenteeism) relations but found little consistency across countries [2,5,6]. An Upstate New York study considered learner absenteeism in relation to building problems and found associations with visible mould, high humidity, poor ventilation, and presence of vermin [14]. These schools had heating, ventilation, and air conditioning (HVAC) systems—none of the schools in our study had such systems. While they also considered building structure characteristics (e.g., floor, exterior wall, and roofs), none of these were found to be associated with learner absenteeism in their study.

### 4.1. Study Limitations

Several factors affect absenteeism other than ambient or indoor classroom temperature, such as menstruation among girls [34], violence and abuse [35], neglect caused by poverty [36], and illnesses and diseases unrelated to temperature exposure, e.g., cancer. These factors, while important, were not considered in the ecological analyses here, and future studies should attempt to do so.

An attempt was made to consider co-variates (including potential confounders and effect modifiers) such as socio-economic status, home conditions, and learner’s reasons for absenteeism by sending home a questionnaire for parents/guardians to complete. Despite the parent/guardian questionnaire response rate being relatively low (as well as many unanswered questions), we have included some of these data to gain insights about the learners for whom data were available.

Parent/guardian-reported questionnaire data revealed that the majority of learners’ households relied on social grants, and few had access to medical aid. These two factors suggest that poverty is high in the communities in which the schools were located. The majority of the included schools were quintile 3 (i.e., no fee-paying schools) and provided a school nutrition programme that gives children one hot meal a day paid for by the government. Many children rely on this meal as their main or only meal of the day.

When parents/guardians were asked what the main reasons were for when the learner was absent, they reported illness and secondly family commitments (although less common). We also considered the impact of school transport, but from the questionnaire, the majority of the learners in the sample lived within one km of their school and therefore likely walked to school. Many reasons other than temperature can influence a learner’s health and well-being, and therefore, this ecological study is only the first step towards understanding the influence of classroom temperature on absenteeism as the outcome of interest.

### 4.2. Lessons Learned

This was the first study of its kind in South Africa, and we noted several issues that should be considered in future studies to build on these baseline results. Since temperatures in South Africa, and globally, are changing, it is important to understand how the school environment may impact learner health and wellbeing outcomes, such as absenteeism.

We installed temperature and humidity loggers at each school. However, it would have been useful to gather rainfall and wind data for each school, too. In this instance, one would need a low-cost weather station located at each school. One could then collect data for each school. In our study, there was only one South African Weather Service station in the study area, and hence, assigning every school the same rainfall and wind data would not have been useful in any analyses. Future studies should include a weather station that measures all meteorological factors that may influence learner absenteeism. For example, a learner may not go to school when it is very rainy and wet. Having local school data about rainfall would allow for such investigations.

In rural (and some urban) schools in South Africa, absenteeism data are typically collected on paper by teachers. The reason for absenteeism is not recorded as part of a follow up when the learner returns to school (unless, for example, in the case of a long illness when a confidential doctor’s note would be provided by the learner to the teacher). If one were to gather the reason for absenteeism for a learner when they returned to work, one would need to ask the teacher to complete a form for each learner; however, teachers have heavy workloads, and this may be too onerous a task to ask of them. One could station a permanent fieldworker at the study for the duration of the study period to conduct these follow-up activities. In this way, the reason for absenteeism could be captured by the fieldworker when the learner returned to school, of course, gathering the reasons within the rights of privacy of information. Data completeness was a challenge when working in rural areas, and alternate methods for capturing data, especially absenteeism records and reasons for absenteeism, should be considered, such as a permanent fieldwork on site. One might also consider using tablets or mobile phones where electricity and internet connectivity are available.

It is also important to consider the timing of exposure to ‘risky’ temperature and absenteeism—a child may experience sitting in a classroom with indoor classroom Tapp levels causing ill health effects and be absent the following day; or a child may wake up and feel outdoor Tapp/temperature levels and decide not to go to school (i.e., be marked absent). In another instance, a child may sit in a cold classroom for three days and on the fourth day does not attend school—possibly due to cold-related illness (although other reasons may exist too). Given the study design, we were not able to ascertain these relations, but future studies may consider doing so by using time-activity diaries. Time-activity diaries can be conducted on paper or using a tablet (with the considerations mentioned above borne in mind); however, they can be onerous on children and participant fatigue may occur. The presence of a permanent school-based fieldworker may assist in increasing the likelihood of participant compliance.

An important consideration in the study of reasons for absenteeism is that multiple factors besides meteorological or climate factors may play a part. Using a questionnaire for parents/guardians can assist with some of these data. In the case of our study, it was likely that given the education level among the parent/guardian population we surveyed, poor literacy may have influenced the completion of the questionnaire and/or the participation in the study. Face-to-face interviews would have helped to resolve this but are costly in a rural setting. The reasons for the latter are the need for fieldworkers who travel to learners’ homes to conduct interviews.

Finally, additional factors such as public holidays and events, and other anomalies should be considered. While we considered school and public holidays, we only became aware of the impact of a public holiday when looking at one specific anomaly: this related to the day after a public holiday when learners may have been away with family for the day and did not return home until the following day, for example. Such intricacies associated with reasons for learner absenteeism would be best understood by social scientists and social anthropologists. Therefore, a large, multidisciplinary research team should be gathered when trying to unravel the effect of temperature on learner absenteeism.

## 5. Conclusions

Large differences of up to 5 °C were seen between indoor classroom and outdoor temperatures, with classrooms being either colder or warmer than the outdoor environment depending on the season. These differences were seen to be related to absenteeism among learners, although several other factors also influence learner absenteeism. Classroom characteristics affected indoor classroom temperatures; hence, thermal comfort should be considered in the design of school buildings for optimal attendance and performance among learners. Our findings have implications for the Department of Education in South Africa and similar settings. There is an obligation to consider temperature and thermal comfort in schools, in terms of design, materials, orientation, ceilings, cooling mechanisms, crowding, shade/greening, early warning systems, etc., given that temperature increases associated with climate change need to be considered now. Furthermore, given possible relationships between temperature and absenteeism, there is also a learning imperative to consider thermal comfort as a fundamental element of school planning and design. These findings support the need for action to climate-proof school classrooms to ensure safe, healthy environments in which learners can learn and work to ensure quality education, and a need highlighted by SDG 4–Quality Education.

## Figures and Tables

**Table 1:** Apparent temperature thresholds and potential health impacts [24].

Symptom Band	US NWS Classification	Apparent Temperature Range (°C)	US NWS Classified “Effect on the Body”
I	Caution	27–32	Fatigue possible with prolonged exposure and/or physical activity
II	Extreme caution	32–39	Heat stroke, heat cramps, or heat exhaustion possible with prolonged exposure and/or physical activity
III	Danger	39–51	Heat cramps or heat exhaustion likely, and heat stroke possible with prolonged exposure and/or physical activity
IV	Extreme Danger	>51	Heat stroke highly likely

**Table 2:** Results of the learner questionnaire completed by parents/guardians for the learners (*n* = 979) who participated in the study during the one-year study period from June 2017 to May 2018.

		Frequency	
Section	Variable	*n*	%
Household characteristics	Anyone with a social grant		
	No	246	25
	Yes	731	75
	Missing	0	0
	Learner has medical aid		
	No	781	80
	Yes	198	20
	Missing	0	0
	Dwelling type		
	Brick house	800	82
	Shack	98	10
	Mud house	76	8
	Missing	0	0
	Primary energy source		
	Electricity	834	86
	Paraffin	60	6
	Gas	63	6
	Wood	20	2
	Missing	2	0
	Type of sanitation		
	Flush toilet	463	47
	Pit latrine	497	51
	Bucket system	5	1
	Missing	14	1
	Water source		
	Tap inside dwelling	460	47
	Tap on stand	392	40
	Communal tap	68	7
	Government tank	28	3
	River	25	2
	Missing	8	1
Learner-related school variables	Numbers of learners attending schools of different quintiles		
	Quintile 1	0	0
	Quintile 2	22	2
	Quintile 3	747	76
	Quintile 4	130	13
	Quintile 5	44	5
	Not specified	36	4
	Distance to school		
	<1 km	568	58
	1–2 km	106	11
	2–3 km	90	9
	3–5 km	137	14
	>5 km	67	7
	Missing	11	1
	School feeding programme		
	No	251	26
	Yes	728	74
	Missing	0	0
	General reasons for absence from school		
	Illness	809	83
	Family commitments	28	3
	Other (undefined)	2	0
	Missing	140	14

**Table 3:** Description of participating schools’ classrooms (*n* = 13) by building characteristics.

Variable	Frequency	
	(*n* = 13)	
	*n*	%
Roof material		
Clay tile	1	8
Corrugated iron	9	69
Asbestos	2	15
Missing	1	8
Ceiling material		
Cement	1	8
Wood	1	8
Ceiling boards	11	84
Floor material		
Wood	4	31
Tiles	1	7
Linoleum/vinyl	8	62
Exterior of school		
Brick	6	46
Plastered brick	2	15
Wood	1	8
Corrugated iron	1	8
Prefabricated walling	1	8
Combination	2	15
Interior of classroom		
Plastered brick	7	54
Corrugated iron	1	8
Unknown	2	15
Combination	3	23
Windows shaded with protection (e.g., awning, tree, curtain)		
No	10	77
Yes	3	23
Temperature control (e.g., mechanical fan, air conditioning, air cooler, whirly roof vent)		
No	8	62
Yes	4	30
Missing	1	8

**Table 4:** Mixed-effects negative binomial model with absenteeism, classroom characteristics, and classroom indoor temperature. A total of 13 schools with complete data and 2302 observations were included in the final model.

Parameter	Est.	*p*-Value	Exp (Est.) %	95% CI%	
Indoor temperature	−0.08	0.052	0.92	0.85	1.00
Wall material					
Brick ^a^	-	-	-	-	-
Prefabricated	−1.34	0.170	0.26	0.04	1.61
Floor material					
Linoleum/vinyl ^a^	-	-	-	-	-
Wood	−1.28	0.126	0.28	0.05	1.44
Tiles	−3.47	0.020 *	0.03	0.00	0.58
Ceiling material					
Cement ^a^	-	-	-	-	-
Wood	2.08	0.188	8.03	0.36	179.18
Ceiling boards	1.02	0.286	2.77	0.43	17.93
Wall material # Indoor temperature					
Prefabricated	0.05	0.240	1.05	0.97	1.15
Floor material # Indoor temperature					
Wood	0.06	0.117	1.07	0.98	1.15
Tiles	0.15	0.045 *	1.16	1.00	1.35
Ceiling material # Indoor temperature					
Wood	−0.11	0.189	0.90	0.77	1.05
Ceiling boards	−0.03	0.513	0.97	0.88	1.07

Note. Est.: Estimate (a positive Est. refers to an increase in learner absenteeism, whereas a negative Est. value refers to a decrease in learner absenteeism); Exp (Est.) % is the incidence risk ratio along with its associated 95% confidence interval; and ^a^ is the reference category. A # shows the interaction between indoor temperature and the respective classroom characteristic. * is the 95% level of significance.

## Data Availability

The data presented in this study are available on request from the corresponding author. The data are not publicly available due to them being a part of a doctoral study and additional articles are still being prepared.
